# Prism adaptation does not change the rightward spatial preference bias found with ambiguous stimuli in unilateral neglect

**DOI:** 10.1016/j.cortex.2010.01.006

**Published:** 2011-03

**Authors:** Margarita Sarri, Richard Greenwood, Lalit Kalra, Jon Driver

**Affiliations:** a MRC Cognition and Brain Sciences Unit, Cambridge, UK; b Institute of Cognitive Neuroscience, University College London, UK; c Regional Neurological Rehabilitation Unit, Homerton University Hospital, London, UK; d Department of Diabetes, Endocrinology and Internal Medicine, Guy's, King's and St. Thomas' School of Medicine, Denmark Hill Campus, London, UK

**Keywords:** Spatial neglect, Prism adaptation, Chimerics, Faces, Spatial bias

## Abstract

Previous research has shown that prism adaptation (prism adaptation) can ameliorate several symptoms of spatial neglect after right-hemisphere damage. But the mechanisms behind this remain unclear. Recently we reported that prisms may increase leftward awareness for neglect in a task using chimeric visual objects, despite apparently not affecting awareness in a task using chimeric emotional faces (Sarri et al., 2006). Here we explored potential reasons for this apparent discrepancy in outcome, by testing further whether the lack of a prism effect on the chimeric face task task could be explained by: i) the specific category of stimuli used (faces as opposed to objects); ii) the affective nature of the stimuli; and/or iii) the particular task implemented, with the chimeric face task requiring forced-choice judgements of lateral ‘preference’ between pairs of identical, but left/right mirror-reversed chimeric face tasks (as opposed to identification for the chimeric object task). We replicated our previous pattern of no impact of prisms on the emotional chimeric face task here in a new series of patients, while also similarly finding no beneficial impact on another lateral ‘preference’ measure that used non-face non-emotional stimuli, namely greyscale gradients. By contrast, we found the usual beneficial impact of prism adaptation (prism adaptation) on some conventional measures of neglect, and improvements for at least some patients in a different face task, requiring explicit discrimination of the chimeric or non-chimeric nature of face stimuli. The new findings indicate that prism therapy does not alter spatial biases in neglect as revealed by ‘lateral preference tasks’ that have no right or wrong answer (requiring forced-choice judgements on left/right mirror-reversed stimuli), regardless of whether these employ face or non-face stimuli. But our data also show that prism therapy can beneficially modulate some aspects of visual awareness in spatial neglect not only for objects, but also for face stimuli, in some cases.

## 1 Introduction

Spatial neglect is a frequent multi-component syndrome following stroke, with the deficits including losses of awareness, orientation and exploration towards the contralesional side of space, which typically cannot be attributed to primary sensory or motor deficits. Neglect patients may fail to acknowledge the existence of contralesional stimuli, and may even neglect contralesional parts of their own body or of mental representations (Mesulam, 1999; Karnath et al., 2002; Driver et al., 2004). When exploring a scene, their eye, body and hand-movements may fail to be directed towards leftward elements (e.g., Farne et al., 2003; Marotta et al., 2003). Neglect is predominantly seen after right-hemisphere damage, most often involving the middle cerebral artery territory (e.g., Karnath et al., 2001, 2004; Mort et al., 2003), although neglect after damage in the posterior (see e.g., Mort et al., 2003) or anterior cerebral artery region (e.g., Klatka et al., 1998) is also possible.

Several attempts to rehabilitate neglect have been made over the last two decades (for reviews see Manly, 2002; Barrett et al., 2006; Luaute et al., 2006), due to the common and highly disabling nature of this syndrome (e.g., Buxbaum et al., 2004; Gillen et al., 2005). Recent efforts to rehabilitate neglect include a promising approach involving adaptation to rightward optical displacement induced by prisms (e.g., Rossetti et al., 1998). The procedure involves a short exposure period (typically lasting only ∼5–10 min) to a prismatic optical shift of 10–15° to the right, combined with a concurrent visuomotor task (usually pointing to visual targets in free vision, while wearing the prisms). Subsequent testing takes place after the prisms have been removed.

Remarkably, this simple, brief and non-invasive technique has now been reported to produce significant improvements in neglect that may generalise across several different aspects, according to numerous studies [e.g., see Rossetti et al., 1998, 2004; Rode et al., 2001; Tilikete et al., 2001; Farne et al., 2002; McIntosh et al., 2002; Maravita et al., 2003; Angeli et al., 2004; Berberovic et al., 2004; Dijkerman et al., 2004; Sarri et al., 2006, 2008; Serino et al., 2007, 2009; Jacquin-Courtois et al., 2008; Saevarsson et al., 2009; Schindler et al., 2009; see also Redding and Wallace, 2006 and Pisella et al., 2006 for recent reviews; but see also Morris et al., 2004; Rousseaux et al., 2006; Nys et al., 2008 for some challenges to the efficacy of prism adaptation (prism adaptation) in neglect]. Improvements have been reported to be relatively long-lasting, for several hours or even days in some cases (e.g., Frassinetti et al., 2002) and possibly much longer after repeated treatment sessions (e.g., Serino et al., 2007, 2009). Reported improvements include reduction of neglect on several traditional paper-and-pencil clinical tests (e.g., line cancellation, line bisection, copying of figures), as well as for activities more relevant to everyday life including postural control (Tilikete et al., 2001) and wheelchair navigation (Jacquin-Courtois et al., 2008). Moreover, the beneficial effects may generalise beyond the visual domain, to include improvements in haptic exploration (McIntosh et al., 2002), tactile extinction (Maravita et al., 2003) and proprioception (Dijkerman et al., 2004), as well as improvements in tasks requiring a verbal rather than spatial motor response, such as object naming (Sarri et al., 2006) and reading (Farne et al., 2002). Finally, prism adaptation has been reported to impact on more abstract levels of spatial representation also, including mental imagery (Rode et al., 2001), and number-line bisection (Rossetti et al., 2004).

**Fig. 1:**
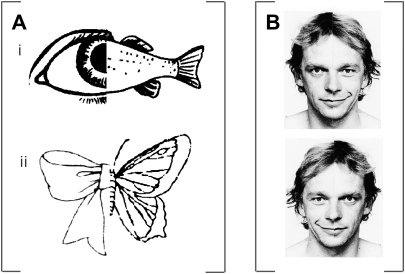
Example stimuli used in Sarri et al. (2006). A. Two examples of chimeric object stimuli used in the chimeric object naming task; note that each chimeric object was presented individually, rather than in a pair as shown for (i) and (ii) here. B. Example of chimeric face pair stimulus used in the chimeric face lateral preference task; neglect patients in this task typically show a right bias by systematically choosing the face with the smiling half on the right side of the display as ‘happier’ (corresponding to the upper stimulus in the pair here); while normals typically show a mild left bias.

In a recent study (Sarri et al., 2006) we reported that prism adaptation (to a 10° rightward optical shift, analogously to the Rossetti et al., 1998 procedure) can improve aspects of perceptual awareness for the contralesional side of some stimuli, despite other suggestions to the contrary (Ferber et al., 2003). Specifically, in the patients studied we found that prism therapy can improve perceptual awareness and explicit report for the contralesional side of *chimeric visual objects* (i.e., stimuli that join together left and right halves of different identifiable objects) in neglect; see Fig. 1A. All three of the participating right-hemisphere stroke patients demonstrated a dramatic increase of awareness for the left (previously neglected side) of chimeric objects following a short adaptation procedure to rightward deviating prisms. We have now replicated these findings in several further patient cases with neglect, all showing similar improvement in explicit naming of the left side of chimeric non-face objects after prism adaptation.

Interestingly though we also found in the same study (Sarri et al., 2006) that the very same prism procedure had no beneficial effect on a task requiring emotional expression judgements for chimeric face stimuli (see Fig. 1B). In this task, the same patients were shown pairs of vertically arranged, identical, left-right mirror-reversed chimeric face stimuli (i.e., joining together left and right halves of the same face posing different neutral or happy expressions) and asked to judge whether the upper or bottom face looked happier. Right-hemisphere damaged patients with left neglect typically select the face that is smiling on the right side of the display (e.g., Mattingley et al., 1993, 1994; Ferber et al., 2003), whereas the opposite tends to apply for normal controls (e.g., Mattingley et al., 1993, 1994; Ferber and Murray, 2005). Prism adaptation did not alter the strong rightward bias or ‘preference’ exhibited by the patients in this task. This latter finding in our three patients (Sarri et al., 2006) was a direct replication of a previously reported single-case study by Ferber et al. (2003), who likewise showed that their patient continued to show a strong rightward bias in the face expression task after prism adaptation (despite an increase of ocular exploration towards the contralesional side in their case).

Thus the apparent discrepancy between the effects of prism adaptation on different chimeric tasks, with benefits being found for identification of non-face chimeric objects (Sarri et al., 2006) yet not for emotional judgements of chimeric face tasks (Ferber et al., 2003; Sarri et al., 2006), still requires explanation. For the existing results, it may be hard to compare directly across tasks that varied both in the nature of the judgement required and in the nature of the stimuli employed. One possibility is that specialized face-processing mechanisms in the brain, as indexed in the Mattingley et al. (1993) chimeric face expression task, may be less influenced by the prism intervention in neglect patients, than for other classes of stimuli. This might conceivably accord with abundant evidence for putatively specialized neural mechanisms for the processing of faces (e.g., see Farah et al., 1995; Kanwisher, 2000; Duchaine and Nakayama, 2005) along ventral pathways, along with other recent suggestions that prism adaptation may primarily affect more dorsal pathways instead (e.g., Dankert and Ferber, 2006). We note also that the judgement required of the chimeric face tasks is based on *emotion* recognition, which might potentially be less influenced by prism therapy than non-affective mechanisms (for evidence on the potentially separate mechanisms supporting recognition of facial identity versus emotion, see e.g., Bowers et al., 1985; Young et al., 1993; and for specialized neural mechanisms for processing of emotional facial expressions see, e.g., Dolan et al., 1996; Winston et al., 2003; Vuilleumier and Pourtois, 2007).

On the other hand, the reported lack of prism effects for the chimeric face task might reflect some particular aspect of the task used, rather than the category of stimulus (i.e., face versus non-face, or affective versus non-affective). Whereas the chimeric non-face object task used by Sarri et al. (2006) ‘explicitly’ tested for awareness of the contralesional space, requiring identification and naming of specific object halves, the chimeric face task of Mattingley et al. (1994), as used by Sarri et al. (2006) and Ferber et al. (2003), is more ‘implicit’ in nature, possibly tapping into a lateral ‘preference’ or bias for one or other side of space, regardless of information content. In the chimeric face task (of judging which face looks happier, the upper or lower) there is in fact no objective correct response, since the two chimeric face tasks are perfect mirror images of each other (see Fig. 1B) and hence objectively contain the same amount of emotional expression.

The present study was designed to explore potential reasons for the apparent discrepancy between the impact of prism adaptation on different measures for neglect, as observed in Sarri et al. (2006). First, we hypothesised that if the lack of a prism effect in the chimeric face expression judgement task is simply due to the special nature of *face* stimuli in general, then prism adaptation should likewise have no effect on neglect for other tasks involving chimeric face tasks. But the lack of a prism effect on the chimeric face expression task might also potentially reflect the ‘emotional’ nature of the task. If so, we would expect a different outcome in a task requiring non-emotional judgements for the same face stimuli, or in a ‘lateral preference task’ employing non-emotional, non-face stimuli. On the other hand, if the lack of prism benefit for the chimeric face expression task is due to the nature of the task used (which can be considered a more ‘implicit’ or ‘indirect’ measure of spatial awareness, since there is no right or wrong answer), then we should find a similar outcome (i.e., no prism benefit) for other tasks of that nature in neglect, even if not using face stimuli. By the same token, we might find a positive impact of prism therapy for tasks employing chimeric face stimuli, but requiring more ‘explicit’ recognition for the left side of the chimeras, by analogy with the chimeric objects studied in Sarri et al. (2006). We thus examined the impact of the prism intervention on neglect performance in tasks employing both face *and* non-face stimuli, for tasks requiring ‘explicit’ or more ‘indirect’ measures of perceptual awareness, in ‘emotional’ or ‘non-emotional’ contexts.

**Fig. 2:**
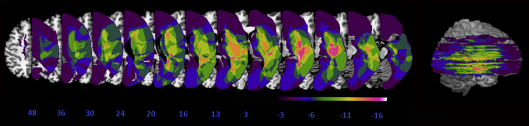
Lesion overlap map summarising the degree of involvement for each voxel in the lesions of all participating patients (except for case TL for which there was no scan available). The range of the colour scale derives from the absolute number of patient lesions involved in each voxel. The map is presented as 2D axial renderings on the MNI ‘representative’ brain, in descending steps. 12 axial slices are shown that correspond to *Z*-coordinates 48, 36, 30, 24, 20, 16, 13, 3, −3,−6,−11 and −16 of the MNI space. The regions of maximal overlap in this group of patients (illustrated here in pink and white), appear to be in the right basal ganglia and white matter underlying the insular cortex and the temporo-parietal junction, areas known to be implicated in the neglect syndrome. These lesion data are presented here solely for summary descriptive purposes.

**Fig. 3:**
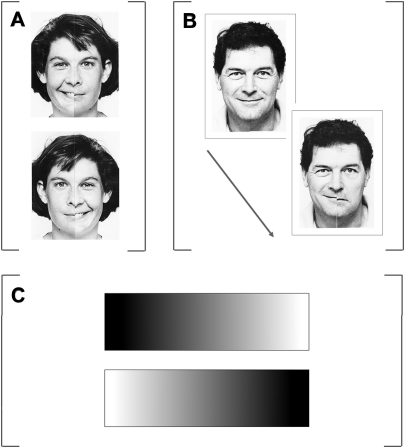
Examples of stimuli used in each experimental task in the present study. A. Chimeric face pair display as used in the chimeric face lateral preference task B. Chimeric and non-chimeric face stimuli used in the chimeric/non-chimeric face discrimination task, each presented singly. C. Pair of greyscale gradients as used in the greyscale gradients lateral preference task.

Here we assessed a new case-series of 11 neglect patients (see Fig. 2 for a summary of their lesions, and the Results section for a summary of clinical details). We first sought to assess any impact of the prism intervention on the chimeric expression lateral preference face task (as previously reported to be absent for 3 cases by Sarri et al., 2006, and for one case by Ferber et al., 2003). The novel manipulation was that we further compared the effect of prism adaptation on neglect in this chimeric face lateral preference task, against its impact on two other tasks. One was a similar spatial ‘preference’ task, with no right or wrong answer, but employing *non-face* stimuli, namely greyscale gradient rectangles (see Fig. 3C). In analogy with the chimeric face preference task, in this greyscale gradient task the patients were presented with pairs of identical left-right mirror-reversed greyscale rectangles, ranging from pure white at one end to pure black at the other end and were asked to indicate which one (upper or lower) seemed ‘darker’ to them. This task has been previously used to assess spatial biases in both normal subjects and neglect patients (e.g., Mattingley et al., 1994, 2004; Loftus et al., 2009). Just like for the chimeric face lateral preference task, neglect patients tend to show a strong rightward bias in this greyscale task and normals tend to show a mild bias towards the left. Of particular relevance here is that this well-established greyscale task should presumably not involve any face-specific or emotional processing mechanisms. The final task implemented here used chimeric face stimuli, but now requiring ‘explicit’ identification of the relationship between the left and right sides of the chimeric face tasks (objective discrimination between ‘chimeric’ and ‘non-chimeric’ face stimuli, see Fig. 3B). Unlike the greyscale or face lateral preference tasks, this task is unambiguous in having a single objectively correct response (rather than merely requiring a choice between left/right mirror-imaged pairs) and in explicitly measuring awareness for the contralesional side, rather than indirectly via spatial preferences. We note also that it does not require any emotional assessment of the stimuli.

If there is something special about prism adaptation effects on face-specific processing mechanisms, we might find a prism benefit on neglect for the greyscale lateral preference task, but not for the other two tasks that do employ faces (expression lateral preference or chimeric versus non-chimeric discrimination). Alternatively, if prism adaptation is ineffective only in tasks that involve emotional processing in particular, we should again expect no prism benefit for the chimeric expression task, but we should find a benefit for the other two tasks (greyscale lateral preference, and chimeric/non-chimeric discrimination of faces), since they do not require emotional processing of the stimuli. Finally, if prism therapy can influence face-related mechanisms, but does not affect spatial preference biases, we should expect no prism benefit in either of the two lateral preference tasks (face expressions or greyscale gradients), yet could potentially find some prism benefit for the chimeric/non-chimeric face discrimination task.

## 2 Methods

### 2.1 Patients

A series of eleven consecutive right-hemisphere stroke patients with left neglect were recruited for this experiment (7 males). All patients had fairly typical lesions and symptoms for right-hemisphere stroke patients with left neglect. See Fig. 2 for a lesion overlap map for our eleven cases (the extent and location of each patient's lesion was defined and visualized using the MRIcro software package Rorden and Brett, 2000; lesions were plotted on 12 axial slices of the T1-weighted template MRI scan from the Montreal Neurological Institute – MNI). All our patients showed neglect on clinical paper-and-pencil measures including the Mesulam cancellation test, a 5-item line bisection task, figure copying and drawing from memory. Diagnosis of left visual neglect involved the fulfillment of at least two of the following criteria: the presence of a minimum 30% omissions on the left side of the page for the cancellation test; a minimum rightward deviation of 12% or more in the line bisection task; omission of left sided elements in the figure copying task; omission of left sided elements in the drawing from memory task. Five out of eleven patients (EY, AK, BH, PH, MM and LG) also presented with complete left homonymous hemianopia as tested on confrontation. See Table 1 for a summary of individual patient details and scores on some paper-and-pencil tasks.

Three of these patients (AK, EY and CO) had already taken part in our previous study (Sarri et al., 2006), but were retested here for the chimeric expression lateral preference task, after a minimum interval of at least one month between testing sessions, to allow within-session comparison with the other tasks. All patients participated in the emotional expressions and the greyscale gradients lateral preference tasks. However, only six patients (EH, AM, PH, EY, LG and MK) were able to participate in the chimeric/non-chimeric face discrimination task. All other patients were excluded from this task as they were found to perform at ceiling-level in this prior to prism adaptation. Please note that in the present study, each patient served as his/her own control (i.e., before versus after prism therapy).

### 2.2 Experimental tasks

#### 2.2.1 Chimeric face lateral preference task

For the chimeric face tasks, 20 pairs of chimeric face tasks were used, adapted from Mattingley et al. (1993). These chimeric face tasks were generated from 10 pictures of 10 different people with a neutral expression, plus 10 pictures of those same people smiling. The photographed faces were divided along the vertical midline, and left and right halves from different photographs of the same person were then juxtaposed in such a way that a smiling half face was on the left and a neutral half face on the right; or vice versa in mirror-image displays. Each chimeric face task subtended approximately 6° × 8°. Chimeric face stimuli were then arranged in vertical pairs, one above the other, so that each pair contained two chimeras of the same person, one neutral in the left half and smiling in the right half, and the other the reverse of this, with vertical position counterbalanced. Thus, the two stimuli arranged vertically were left/right mirror images of each other; see Fig. 3A for examples. The patients were told that they would be shown a series of faces in pairs and that for each pair they had to choose the one they thought “looked happier”. Patients were shown the 20 pairs of chimeric face tasks in turn and asked to indicate verbally for each display whether the upper or lower member of each pair looked happier, just as in Mattingley et al. (1993), Ferber et al. (2003) and Sarri et al. (2006). The stimuli were placed in front of the patients on a table, centred on the mid-sagittal plane of their head and trunk, and remained in view until the patients gave a response, without any time limit.

#### 2.2.2 Greyscale gradients lateral preference task

For the gradients task, 20 pairs of greyscale gradients were constructed analogously to those in Mattingley et al. (1994). 10 pairs of greyscale gradient rectangles, consisting of a continuous scale of grey shades varying from absolute white at one end to absolute black at the other end were produced and printed on A4 sheets of paper. Each pair consisted of two rectangles, one being the mirror-reversed image of the other, one presented above and one below (see Fig. 3C). Each rectangle was bound by a .5 mm black outline. The two rectangular strips varied in length from 10–20 cm (thus subtending approximately 15–28°), in increments of 1.5 cm and were kept at a constant height of 5 cm (approximately 4°). The two strips were always kept apart at a constant vertical separation of 2 cm. These 10 pairs were then mirror reversed to produce another 10 pairs. Patients were presented with all 20 pairs of identical but mirror-reversed greyscale gradient rectangles and asked to report verbally whether the upper or lower member of each pair looked darker (by saying ‘top’ or ‘bottom’), as in Mattingley et al. (1994). The stimuli were placed in front of the patients on a table, centred on the mid-sagittal plane of their head and trunk and remained in view until the patients gave a response, without any time limit.

#### 2.2.3 Chimeric/non-chimeric face discrimination

For the explicit chimeric/non-chimeric face discrimination task, 20 non-chimeric (‘real’) and 20 chimeric face stimuli were used, taken and adapted from Mattingley et al. (1993). The chimeric face stimuli were constructed from half-parts of the 20 non-chimeric face stimuli. The construction of the chimeric face stimuli was identical to the one described for the chimeric face lateral preference task. Each face stimulus subtended approximately 12° × 16° and unlike the emotional judgement task, where faces were presented in pairs, each face here was now presented individually. See Fig. 3B for an example of a non-chimeric and a matched chimeric face stimulus (note that this illustration depicts two potential successive trials, although in practice the face on one trial was unlikely to relate to that on the next).

All 20 chimeric face stimuli were intermingled with the 20 non-chimeric face stimuli, so a total of 40 individual face stimuli were presented in random sequence. Each stimulus was presented briefly in the centre of a computer monitor for approximately 2.5 sec (presentation time ranging between 2–3 sec, and adjusted for each patient to match the minimum time required for the patient reliably to give a response, then kept constant before and after the prism adaptation procedure). Patients were told that they would be shown a series of pictures of faces, some of which would be ‘real’ pictures of people with neutral or happy expression and some of which would be ‘chimeric’, i.e., having two halves, depicting the same person but with a different emotional expression on the two halves (see Fig. 3B). Patients were then shown an example of each stimulus type on paper, and the experimenter made sure that the patient understood the difference between the two types of stimuli, drawing their attention to differences between the two sides within the chimeric if required, and checking that the patient could then verbally describe those differences correctly. The patients were then positioned at a distance of ∼55 cm from the computer monitor and were asked to indicate verbally whether each face stimulus was ‘real’ or ‘chimeric’. Responses were recorded by the experimenter and performance scored in terms of accuracy.

### 2.3 Procedure

#### 2.3.1 Experimental procedure

Patients were given all three tasks (i.e., chimeric face task lateral preference task, gradients lateral preference task and chimeric/non-chimeric face discrimination task) before and immediately after the prism adaptation procedure. The order of stimuli presentation was randomised both before and after the prism adaptation procedure, for all tasks and for all patients, as was task order. For completeness, patients also underwent quick standard measures of neglect, completing 3 line bisections (180 mm lines) and 5 subjective straight-ahead pointing movements (with right hand and eyes closed) both before and after the adaptation procedure (with the exception that if no clear neglect was shown on either or both of those measures prior to prisms, the particular measure was not repeated after prisms). The order of task presentation was random, but was held constant before and after prism adaptation for each patient. No feedback was provided during testing.

#### 2.3.2 Prism-adaptation procedure

For the prism adaptation procedure the patients sat at a table. During adaptation they wore base-left wedge prisms that induced a 10° optical shift to the right. The adaptation to prisms was accomplished by having the patients perform 60 repeated pointings with their right hand to two targets placed on a table, 10° to the left or right of the centre of their mid-sagittal plane, at a distance of ∼55 cm from their trunk, in a randomly intermingled sequence. Patients were instructed to make fast movements to the targets and then return their arm to the initial starting position on the table by their trunk centre. The initial position of their arm was occluded by a horizontal board, obscuring approximately 25% of the distance between the patient and the targets in accord with the usual method employed by Rossetti and colleagues (e.g., Rossetti et al., 1998) in their pioneering work on prism adaptation in neglect. Hence patients could see their arm only after initiating a movement towards their target, but had closed-loop visual feedback for any terminal errors, thus inducing corrections and adaptation to the prismatic deviation. Total exposure to the prisms was approximately 10 min for each patient, and the prisms were then removed prior to immediately retesting patients on all experimental tasks.

#### 2.3.3 Measurement of prism adaptation after-effects using open-loop pointing

To obtain a measure of prism adaptation success, an additional open-loop (i.e., arm unseen) pointing task was used both before and after prism adaptation, to allow measurement of the expected visuo-manual prismatic after-effect. For this task patients were asked to point several times to a single target (a red dot) placed at the centre of their mid-sagittal plane at a distance of 55 cm, with their right hand, both before and after the prism adaptation procedure. Vision of the hand was completely obscured throughout this aspect of the procedure via an occluding surface placed above the arm. Each patient made 10 open-loop pointings before the adaptation procedure, plus 10 immediately after removing the prisms, to assess whether exposure to rightward shifting prisms had induced the expected (leftward) prism after-effect (as would be found in normals; see also Sarri et al., 2008).

## 3 Results

All eleven patients showed the expected leftward shift in open-loop pointing after exposure to prisms (i.e., a prism after-effect), indicating that the adaptation procedure was successful for all. The mean pointing deviation away from the physically central target after the prism adaptation procedure was 3° (SD = 2.4°) towards the left. This mean leftward deviation in pointing, after the adaptation procedure, was significantly different [*t*(10) = −12.1, *p* < .0001] from the slight tendency for rightward deviation observed before the prismatic procedure (mean .9° rightward, SD = 2.5°). On an individual level, the difference between the pre- and post- adaptation open-loop pointing error was again significant for all patients (*p* < .05). Thus all patients showed significantly more leftward deviation in open-loop central pointing after exposure to the rightward deviating prisms (mean = 3.9°, SD = 1.1°), indicating successful adaptation to the prism-induced optical displacement.

We also found significant improvement after the adaptation procedure for the two standard clinical measures of neglect assessed pre- and post-prisms here. Patients showed a significant change in their subjective straight-ahead pointing [*t*(10) = 9.54, *p* < .001], pointing closer to their ‘true’ straight-ahead midline after prism adaptation (mean deviation error to the left = 1.4°, SD = 5.6°) as opposed to before prisms when they showed a clear rightward deviation (mean = 6.2°, SD = 4.2°). Similarly, for the 7 patients in whom we obtained both pre- and post-prism line bisection data, there was a significant overall improvement in this task post-adaptation. The mean bisection error was 24.7 mm (SD = 10.7 mm) to the right of true centre before prism adaptation, compared to 14.2 mm (SD = 7.8 mm) after prism adaptation [*t*(6) = 7.26, *p* < .001]. Three further patients initially showed no clear neglect for line bisection immediately prior to prisms (i.e., did not meet our criterion of a minimum 12% deviation to the right), so did not undergo line bisection after prisms, while in a final case it was not possible to obtain pre- and post-prism line bisection within their available time, given the need to run all of the other tasks pre and post.

Taken together, the available data on open-loop pointing (for all patients), subjective straight-ahead pointing (again for all patients) and line bisection (available pre- and post for 7 of the 11 patients) clearly show that our prism intervention was effective, both in inducing the usual adaptation after-effect (for open-loop pointing) and also a significant amelioration of neglect on standard quick clinical measures (for subjective straight-ahead and line bisection). Thus, when turning to consider the experimental tasks below, we can already be reassured that the prism intervention was successfully implemented.

### 3.1 Chimeric face lateral preference task

**Fig. 4:**
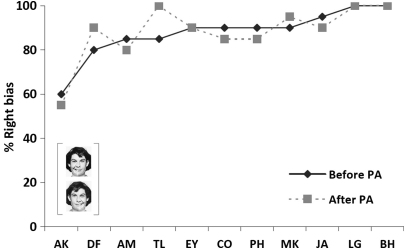
Percentage of right-smiling faces chosen before and after the prism adaptation procedure in the chimeric face lateral preference task, for each individual patient. Patients are ordered along the *x*-axis in terms of the rightward bias demonstrated in this task pre-adaptation (with less bias pre-prisms towards the left of the *x*-axis). Note the clear null effect of prism adaptation on this task irrespective of the amount of initial bias demonstrated.

Before prism adaptation, all eleven participating patients showed a strong bias favouring the right side of chimeric face tasks when making forced-choice lateral preference judgements based on emotional expression, with the exception of AK who again performed at chance level (see also Sarri et al., 2006). Before prism adaptation, patients chose on average the face with the smiling half on the right side of the display as being the ‘happiest’ in 88% of the pairs presented (i.e., mean rightward choice out of the 20 pairs was 17.5, with SD = 2.2). The corresponding mean percentage of right-smiling faces chosen after prism adaptation was again 88% (mean = 17.6, out of the 20 pairs, with SD = 2.6), i.e., identical to the pre-adaptation bias demonstrated in this task, leading to no significant impact of prisms [*t*(10) = −.2, *p* = .8, n.s.]. Thus, the prism intervention was again found to have absolutely no impact on performance in this task for any of the patients tested here, none of whom showed a significant impact of prisms on their lateral preferences for emotional expression. This replicates the results of Sarri et al. (2006) but now in a much larger series of patients, and again in accord with Ferber et al. (2003). See Fig. 4 for individual results.

### 3.2 Greyscale gradients lateral preference task

**Fig. 5:**
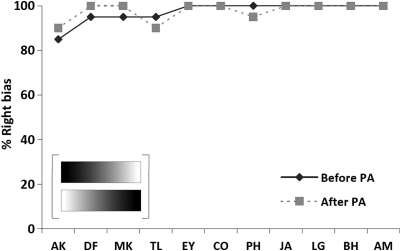
Percentage of gradients chosen as ‘darker’ that had their darker side on the right, before and after the prism-adaptation procedure in the greyscale gradients lateral-preference task, for each individual patient. These data indicate no effect of prism adaptation on the very strong rightward bias in this task, irrespective of the degree of initial bias demonstrated. Patients are ordered along the *x*-axis in terms of the bias demonstrated in this task pre-adaptation.

An analogous pattern was observed for the greyscale gradients lateral preference task. Before prism adaptation, all eleven participating patients showed a very strong bias for their judgement to reflect the right side of the greyscale gradients, which was even stronger than the bias observed for the chimeric face task described above. Even patient AK who did not show a rightward bias in the face expression task (choosing 12/20 faces with the smiling face on the right before prisms, and 11/20 after prisms), demonstrated a strong rightward bias in the gradients task (showing a preference for gradients with the dark side on the right in 17/20 pairs before and 18/20 pairs after prisms). Before prism adaptation, the mean choice of the gradient with the dark side on the right as the ‘darker’ was 98% (mean 19.5 out of 20 pairs, with SD = .9). The corresponding percentage after prism adaptation was again 98% (mean = 19.5 out of 20, with SD = .8). Similarly to the results for the chimeric face lateral preference task, prism intervention was thus found to have no impact whatsoever on lateral preferences in the greyscale gradients task [*t*(10) = 0, *p* = 1, n.s.] and this was true for all the individual participating patients, none of whom showed an individually significant impact of prisms in this task; see Fig. 5.

Thus, for both the chimeric face expression and greyscale gradients lateral preference tasks, all patients showed strong left neglect, manifested as expression or darkness judgements (respectively) being pathologically based on just the right side of the stimuli, unlike the normal tendency for the left side to predominate slightly for both the face task (cf. Levy et al., 1983; Luh et al., 1991; Mattingley et al., 1993, 1994) and the greyscale gradients task (Mattingley et al., 1994; Nicholls et al., 2004, 2005) in neurologically healthy subjects. Indeed all of our neglect patients fell well outside the normative range for these particular tasks (see Mattingley et al., 1994), with the sole exception of patient AK in the chimeric face expression task (see also Sarri et al., 2006). But the main point for present purposes is that the patients' performance for both these lateral preference tasks was completely *unaffected* by prism adaptation (see Figs. 4 and 5).

### 3.3 Chimeric/non-chimeric face discrimination

Turning to the chimeric/non-chimeric face discrimination task, all six participating patients showed signs of neglect in this task before the prism adaptation procedure, failing to classify 40% or more of the chimeric face tasks presented as such. In particular, patients tended to erroneously classify ‘chimeric’ faces as ‘real’, presumably failing to notice any differences in emotional expression between the left and the right halves of the chimeric face tasks, due to their left neglect. By contrast they were mostly accurate at classifying the non-chimeric, ‘real’ faces as such. Specifically, EY classified correctly only 20% of the chimeric face tasks presented (erroneously classifying 80% of the chimeric face tasks presented as ‘real’), whereas she correctly classified 85% of the ‘real’ faces. AM correctly classified 60% of the chimeric face tasks and 80% of the ‘real’ faces; PH correctly classified 40% of the chimeric face tasks and 60% of the ‘real’ faces; BH correctly classified 40% of the chimeric face tasks and 80% of the ‘real’ faces; LG correctly classified 32% of the chimeric face tasks and 67% of the ‘real’ faces; and MK correctly classified 5% of the chimeric face tasks and 95% of the ‘real’ faces, all prior to prisms.

**Fig. 6:**
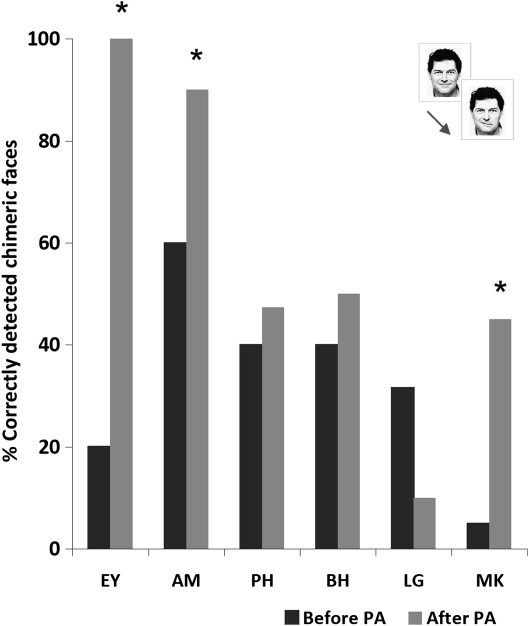
Percentage of chimeric faces correctly classified as such, before and after the prism adaptation procedure, in the chimeric/non-chimeric face discrimination task, for each individual patient. Asterisks mark those patients who showed an individually significant improvement after prisms.

Following prism adaptation EY, AM and MK showed a significant improvement in this task, whereas the performance of PH, BH and LG remained unaffected (see Table 2 and Fig. 6 for individual patient performance), as revealed by chi-square tests performed for each individual patient. After the prism adaptation procedure EY, AM and MK all showed a substantial improvement in classifying the ‘chimeric’ faces correctly [for EY, *χ*^2^(1) = 26.7, *p* < .001; for AM, *χ*^2^(1) = 4.8, *p* < .02; for MK, *χ*^2^(1) = 8.5, *p* < .005], while at the same time their relatively good performance in identifying the ‘real’ faces remained statistically unaffected [for EY, *χ*^2^(1) = 1.3; for AM, *χ*^2^(1) = .78; for MK, *χ*^2^(1) = 3.1; all *p* > .05]. By contrast, the performance of PH, BH and LG in classifying both the chimeric [for PH *χ*^2^(1) = .10; for BH *χ*^2^(1) = .40; for LG *χ*^2^(1) = 2.5; all *p* > .05] and the non-chimeric [*χ*^2^(1) = .107; for BH *χ*^2^(1) = .78; for LG *χ*^2^(1) = 1.9; all *p* > .05] faces remained unaffected by the prism adaptation procedure.

**Fig. 7:**
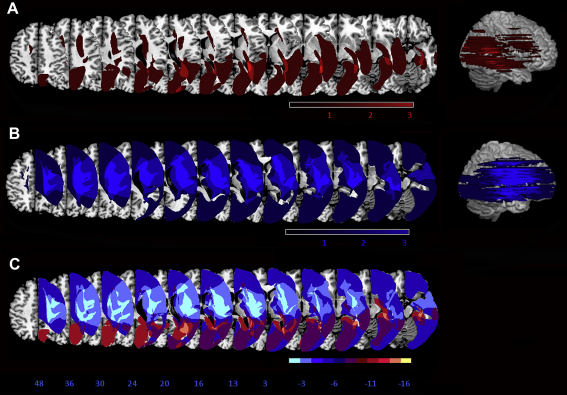
Summary lesion information for non-improved versus significantly improved patients, in terms of the impact of prism exposure on left neglect in the chimeric/non-chimeric face discrimination task. Given the low number of patients in either subgroup (*n* = 3), this lesion information is presented here solely for exploratory descriptive purposes. A. Improved patients (*n* = 3). Overlap map showing the degree of involvement for each voxel in the lesions of the improved neglect group, normalized to the MNI template. The map is presented as axial renderings on the MNI ‘representative’ brain, in descending steps. 12 axial slices are shown that correspond to *Z*-coordinates 48, 36, 30, 24, 20, 16, 13, 3, −3, −6, −11 and −16 of the MNI space. The range of the colour scale derives from the absolute number of patient lesions. B. Overlap map for the non-improved neglect patients (*n* = 3). C. Non-improved minus improved patients. Contrast map showing the relative involvement (bins of 16.67%, apart from the purple shading which represents −16.67% through to +16.67%) of each voxel in the lesions of the non-improved patient group minus the improved patient group. The colour scale covers a range of voxel involvement in the two lesion groups, from involvement in the non-improved neglect group only (light blue) to involvement in the improved neglect group only (light red).

We were encouraged by reviewers to conduct an exploratory assessment of whether lesion details and/or clinical factors might potentially distinguish those patients who clearly benefited from the prism procedure in the chimeric/non-chimeric discrimination task (cases EY, AM and MK) from those who did not (PH, BH and LG), despite the low group sizes. As noted earlier, the extent and location of each patient's lesion was defined and visualized using the MRIcro software package (Rorden and Brett, 2000; www.mricro.com) and plotted on 12 axial slices of the T1-weighted template MRI scan from the Montreal Neurological Institute. A lesion subtraction (see Karnath et al., 2001; Mort et al., 2003), contrasted the lesions of patients who did not show an improvement (PH, BH, LG, see Fig. 7A) versus those who did (EY, AM, MK, see Fig. 7B), to provide a descriptive overview of any differences (see Fig. 7C). This descriptive approach revealed that patients who did not show an improvement tended to have more anterior lesions. Moreover their lesions were larger (mean = 269 cc, SD = 173 cc) than the lesions of patients who did show a prism-induced improvement (mean = 74 cc, SD = 49 cc). Indeed we found a significant negative correlation between lesion size and improvement (post- versus pre-prism performance) in the chimeric/non-chimeric face discrimination task [rho(4) = −.886, *p* = .02], despite the small set of six cases in this particular task. Patients with larger lesions showed smaller prism-induced improvement in this task. The relatively small sample of patients meant that formal voxel-based assessment of any lesion differences (e.g., Bates et al., 2003) was inappropriate (see Medina et al., 2009). Future work on the lesion anatomy of patients which may or may not benefit from prism therapy (see also Sarri et al., 2008) will require larger groups.

Reviewers also encouraged us to undertake exploratory consideration of whether clinical factors such as age, time post stroke and neglect severity on standard measures may relate to any prism impact on the chimeric/non-chimeric face discrimination task. A full assessment of this would again require a much larger sample, in future work. Here we found no significant (or approaching significant) correlations with the prism impact on the chimeric/non-chimeric face discrimination task, for any of these clinical factors. Nevertheless, with future research in mind, it may be worth noting that all patients who showed a prism-induced improvement in the present task were within one and five months post onset, while patients who did not show an improvement typically had an earlier stroke (see Table 1). Moreover, those patients who did not show any significant improvement all had hemianopia, whereas only one out of the three patients who did show a significant improvement had hemianopia.

For present purposes our focus was not so much on identifying which patients may benefit from prism adaptation, as on the nature of the tasks which may or may not benefit. The most important outcome from the chimeric/non-chimeric face discrimination task is simply to show that prism adaptation can improve awareness for the left side of *face* stimuli in at least some cases. Although we found this positive effect reliably only in three out of six of the patients tested here (those who tended to have smaller lesions, and be within five months of stroke onset), the unequivocal improvement in EY, AM and MK's performance provides an existence proof that prism adaptation *can* in principle improve awareness for the left side even of face stimuli, at least in tasks that require explicit detection of differences (in this case emotional expression differences) between the left and the right side of a face stimulus.

## 4 Discussion

Our previous work (Sarri et al., 2006) had reported that while prism therapy may apparently have no effect on neglect patients' awareness for the contralesional side of chimeric face tasks, when measured by forced-choice spatial preference judgements of emotional expression (in which neglect patients pathologically favour the right side of chimeric face tasks, see also Ferber et al., 2003), it can nevertheless significantly increase awareness for the left side of chimeric non-face objects. In the present study we explored potential reasons for the apparent failure of prism adaptation to alter the systematic rightward bias demonstrated by neglect patients in the chimeric face lateral preference task, despite the beneficial effect it has been shown to exert on many other aspects of neglect to date (e.g., see Rossetti et al., 1998, 2004; Rode et al., 2001; Tilikete et al., 2001; Farne et al., 2002; McIntosh et al., 2002; Maravita et al., 2003; Angeli et al., 2004; Berberovic et al., 2004; Dijkerman et al., 2004; Pisella et al., 2006; Sarri et al., 2006, 2008; Serino et al., 2007, 2009; Jacquin-Courtois et al., 2008; Saevarsson et al., 2009; Schindler et al., 2009) and despite the improvement shown in the chimeric non-face object task (Sarri et al., 2006). Specifically, we sought to determine whether the apparently null effect of prism adaptation on the chimeric face task (Ferber et al., 2003; Sarri et al., 2006) could be due to the nature of the stimuli or the nature of the task used. To address these issues, the effect (or lack thereof) of prism adaptation on the chimeric face expression judgement task was compared here with the impact of prisms on a logically similar lateral preference task but now employing non-face, non-emotional stimuli (greyscale gradients); and with the impact on a different task using the same face stimuli again, but now providing a more direct or ‘explicit’ measure of contralesional awareness, having a right versus wrong answer, and requiring no emotional judgement on the stimuli, but simply a judgment of whether they were chimeric or not.

The results replicated those of Sarri et al. (2006) and confirmed previous findings (Ferber et al., 2003) in a new sample of eleven patients, showing persisting, unaltered ipsilesional biases after prism adaptation in the chimeric face lateral preference task, which required forced-choice spatial preference judgements of emotional expression. A strong initial preference bias was found in ten out of eleven patients tested here (all except AK) pre-adaptation, who based their emotional expression judgements predominantly on the right side of the chimeric face stimuli. As also suggested by previous findings (Ferber et al., 2003; Sarri et al., 2006), this lateral bias remained totally unaffected in all patients (even the atypical case of AK also showed no prism impact), after a successful adaptation period to rightward deviating prisms. Moreover, the lack of any prism impact on the face expression lateral preference task contrasted with the clear and significant prism impact on open-loop pointing, and also with the beneficial impact on subjective straight-ahead and line bisection, for which neglect in our patients was clearly reduced by the prism intervention. Thus the lack of a prism impact on the lateral preference face task cannot be due to any overall ineffectiveness of our prism manipulation per se.

Importantly, we also found here an analogous pattern for a similar but non-face, non-emotional lateral preference task requiring darkness judgements for pairs of greyscale gradient rectangles. This task is logically similar in nature to the chimeric face lateral preference task, in also being an ‘implicit’ or indirect measure of perceptual awareness, having no right or wrong answer, while measuring a preferential choice between identical but left-right mirror-reversed stimuli. But they key point for present purposes is that the greyscale task utilized non-face, non-emotional stimuli. In accord with Mattingley et al. (1994) we found that the pre-adaptation rightward bias exhibited by the patients in this task was even more robust than that observed for the lateral preference task with chimeric face tasks. The eleven participating patients chose the gradient with the darker side on the right on average in 98% of trials (as opposed to an average of 88% rightward preferences in the chimeric face task). This very strong rightward bias in the gradients task remained fully present and totally unaffected after the prism adaptation procedure, similarly to the results found for the lateral preference task with chimeric face tasks. Although the 98% bias might be considered as so strong that it represents a ‘ceiling’ or ‘floor’ effect, note that there was in fact plenty of room for the bias to be reduced by prism therapy, yet no benefit of prisms was found on the preference tasks.

Finally, we report here an initial existence proof for a positive effect of prism adaptation (for some patients at least) on a different task employing chimeric face tasks, suggesting that it is possible to improve perception for the contralesional side of *face* stimuli with prism adaptation to some extent, in at least some cases. Using a simple task requiring explicit discrimination of the ‘chimeric’ or ‘non-chimeric’ nature of face stimuli (the same face stimuli as used in the lateral preference task, but now presented individually), we found a tendency for neglect patients to report ‘chimeric’ faces as ‘non-chimeric’, presumably due to neglect for the left half leading to a failure to notice the difference between left and the right halves. Prism adaptation had a significantly positive effect on performance in this particular task, in three out of six cases tested. The patients who did not show this prism-induced improvement tended to have larger lesions (which also appeared to be more anterior, on a descriptive lesion subtraction), although any exact relation to lesion anatomy would require further study in a larger group. But for present purposes, the key point is simply that adaptation to right-shifting prisms can substantially improve visual awareness even for the contralesional side of chimeric face tasks, in at least some patients with left neglect after right-hemisphere damage, depending on the task employed. This finding further indicates that the lack of any prism effect whatsoever on patient performance in the two lateral preference tasks did not merely reflect a general failure of our prism adaptation procedure to produce neglect-related benefits. This point received further convergent support from the significant beneficial effects of our prism intervention on line bisection and subjective straight-ahead pointing, two commonly used clinical measures for assessment of spatial neglect.

Taken together, the present results suggest that prism adaptation may not be effective in changing rightward biases in neglect for lateral preference tasks (see Mattingley et al., 1993, 1994) in which patients are required to make ambiguous choices with no right or wrong answer. Although prism adaptation has been shown to improve performance for the left side of space in numerous aspects of neglect (see reviews by Pisella et al., 2006; Redding and Wallace, 2006) and to increase awareness for the left side of non-face objects in neglect patients (as demonstrated in Sarri et al., 2006) it appears ineffective for lateral preference tasks, possibly irrespective of the type of stimulus used (as shown here for both chimeric face expressions and greyscale gradients). In fact Mattingley et al. (1994, 2004) have shown that performance in these lateral preference tasks does not correlate with other classical tests of neglect (specifically not with cancellation or line bisection in their data) and can be present in patients with unilateral brain damage even in the absence of neglect (see also Peers et al., 2005, and Habekost and Rostrup, 2006, for further demonstrations of similar spontaneous attentional lateral biases in patients with unilateral damage without clinical signs of neglect). Mattingley et al. (1994) reported that although patients' ability to reorient attention contralesionally at will may recover relatively quickly, more subtle ipsilesional attention biases–as potentially measured by lateral preference tasks may be relatively persistent. Thus the lateral preference tasks may tap into a potentially distinct and dissociable deficit involving a ‘chronic’ bias towards the right, which might dissociate from a deficit in controlled shifts of attention towards the contralesional side. In our own data here, five patients (AK, CO, DF, JA and TL) performed at ceiling level in the chimeric/non-chimeric face discrimination task even prior to prisms, implying that these patients could to some degree still become aware of the left side of chimeric face tasks when encouraged by the task. Yet these cases all still showed a strong rightward bias when required to make preference judgements between otherwise equivalent mirror-reversed stimuli, potentially lending further support to the idea of a dissociable deficit underlying lateral preference tasks.

Since rightward biases in lateral preference paradigms can be found even in patients with no other signs of clinical neglect and no frank deficits of perceptual awareness for the contralesional side (see Mattingley et al., 1994, 2004; Habekost and Rostrup, 2006), this might imply that such spatial preferences need not reflect explicit awareness for the contralesional space per se. Instead the lateral preferences may provide a more indirect or implicit measure of any difference in ‘salience’ for the stimuli on either side (e.g., Mattingley et al., 2004). If so, this might be reconciled with prisms on the one hand having some impact on awareness for the contralesional side (as in Maravita, et al., 2003; Sarri et al., 2006; and for half of the present cases in our chimeric/non-chimeric face discrimination task here); yet while on the other hand still having no impact on an implicit bias in salience, as revealed by the lateral preferences. An alternative perspective (e.g., Dankert and Ferber, 2006) is that prism adaptation may primarily affect dorsal pathways concerned with visuomotor behaviour, rather than perceptual awareness per se (see also Ferber et al., 2003). While this remains an intriguing possibility, from our perspective it would not readily explain why prism adaptation can apparently affect perceptual awareness itself for at least some measures of neglect (e.g., see Maravita et al., 2003; Sarri et al., 2006), as also for those cases who showed a benefit after prism adaptation for the explicit chimeric/non-chimeric face discrimination task here. Finally one has to acknowledge the possibility that lateral preference tasks may somehow just be less sensitive to prism benefits in general. However arguing against this is a recent study in normals, showing that the small lateral preferences for greyscale gradients in neurologically healthy subjects can be influenced to some extent by prism interventions for the intact brain (Loftus et al., 2009).

A recent study by Nijboer et al. (2008) found that prism therapy in neglect patients benefited ‘endogenous’ spatial attention (directed voluntarily by a centrally presented symbolic cue) but not ‘exogenous’ spatial attention (directed in a bottom-up manner, by stimulus salience), when studied in spatial cuing paradigms. An impact of prism therapy upon endogenous spatial attention but not exogenous spatial attention in neglect might in principle explain why some tasks but not others benefit from the prism intervention for such patients. In particular, the spatial imbalance revealed by lateral preference tasks (such as the face expression or greyscale paradigms used here) might potentially be determined primarily by pathological spatial changes in the stimulus salience that drives exogenous attention. If so, then given Nijboer et al. (2008) one could predict that the lateral preferences would unaffected by prism adaptation in neglect patients, exactly as we found so clearly for all our cases here.

As pointed out by a reviewer, further potential differences between the tasks found here to be affected or unaffected by prism adaptation in neglect may include variations in attentional load. For instance, the two preference tasks here required a choice between upper and lower stimuli, whereas the chimeric/non-chimeric discrimination task presented just one stimulus at a time (see Fig. 4). To accommodate the present data, any interpretation in terms of load would lead to the testable new hypothesis that the benefits of prism therapy for neglect might be more pronounced for situations with lower attentional load, as might be systematically tested in future research. This could have clinical implications if so, since it is typically high- rather than low-load situations that lead to the most pronounced problems for neglect patients (e.g., see Vuilleumier et al., 2008; Sarri et al., 2009). A further difference between the present tasks pointed out by a reviewer is that the chimeric/non-chimeric discrimination task in particular may ‘cue’ patients to consider both sides given the task requirements. That could potentially explain why some of our patients were unimpaired on this task prior to prisms. On the other hand, we note that the task requirements themselves were held constant pre- and post-prisms, whereas our main focus was on post- versus pre-prisms differences here, i.e., on benefits due to the prism intervention.

A further interesting issue for future research may be to compare the impact of prisms on the different tasks employed here in neglect at various delays after the prism intervention. One intriguing aspect of the classic prism neglect study by Rossetti et al. (1998) was that some aspects of performance were more improved 2 h after prism exposure than immediately after (see also Hatada et al., 2006), whereas here we only tested immediately after. On the other hand, most studies reporting beneficial impact of prisms on neglect have found some benefit immediately after the adaptation procedure (e.g., Rossetti et al., 1998; Rode et al., 2001; Pisella et al., 2002), whereas there was none here for the lateral preference tasks, in any of our eleven cases.

A full understanding of the reasons for prism adaptation benefiting certain tasks or patients but not others (see also Dijkerman et al., 2003; Morris et al., 2004; Rousseaux et al., 2006; Nys et al., 2008; Sarri et al., 2008) will be important not only for understanding the underlying mechanisms, but also for optimising prism adaptation as a potential rehabilitation tool for neglect. While such understanding is not yet complete, we hope the presented results can contribute to it. What we found was a clear dissociation between spatial preference tasks on the one hand which are unaffected by prism adaptation (and may tap into implicit lateral preferences determined by spatial distortions in salience); versus more traditional assessments of neglect (including line bisection and the subjective straight-ahead) that clearly did benefit.

## Figures and Tables

**Table 1:** Summary of individual patient details and scores in diagnostic tests. Notes: Cancellation: total number of targets cancelled on the left (L) and right (R) side of the page (out of 30 on each side) in the Mesulam shape cancellation task; Line bis. %: average percentage of deviation (positive values indicate rightward deviation) from the objective midline in line bisection; Hemianopia: presence (Yes) or absence (No) of hemianopia; TPO: Time post onset of stroke, given in months; ACA, MCA and PCA: anterior, middle and posterior cerebral artery; visualextinction: presence (Yes) or absence (No) of visual extinction on confrontation in patients with intact visual fields; L somatosensory loss: clinical diagnosis of left unilateral somatosensory loss based on confrontation and self report; ‘–’ indicates non-applicable or non-available data for a particular case, each case identified by initials in leftmost column.

Patient	Sex	Age	Handed ness	Hemianopia	TPO (months)	Lesion size (cc)	Pathology and lesion site
EY	F	74	L	Yes	5	99	R parieto-occipital infarct (PCA/MCA ‘watershed’)
AK	M	64	R	Yes	7	62	R haemorrhage affecting external capsule, claustrum and internal capsule
CO	F	57	R	No	9	86	R frontal, basal ganglia and insular MCA infarct
BH	F	59	R	Yes	20	194	R subarachnoid and MCA haemorrhage affecting temporal and inferior parietal cortex
AM	M	67	R	No	1	105	R temporal and parietal MCA infarct
PH	M	51	R	Yes	12	146	R intracerebral subarachnoid haemorrhage affecting basal ganglia and temporo-frontal white matter
DF	M	72	R	No	175	155	R frontal and parietal MCA infarct
JA	M	69	R	No	2	89	R parietal and occipital infarct
TL	M	56	R	No	4	–	R ACA infarct
MK	M	53	R	No	2	17	R temporal and parietal MCA infarct
LG	F	23	R	Yes	6	467	R frontal, temporal and parietal MCA infarct

Patient	Mesulam cancellation	Line bis. %	Omissions in figure copying	Omissions in drawing from memory	Visual extinction	L somatosensory loss	L motor impairment
EY	L: 9, R: 22	40	Yes	Yes	–	No	No impairment
AK	L: 4, R:21	13	Yes	Yes	–	–	Mild hemiparesis
CO	L: 7, R: 26	23	No	No	Yes	No	Severe hemiparesis
BH	L: 2, R: 29	54	No	No	–	–	Hemiplegia
AM	L: 23, R: 29	85	No	No	Yes	Yes	Mild hemiparesis
PH	L: 0, R: 10	9	No	No	–	Yes	Severe hemiparesis
DF	L: 14, R: 29	8	Yes	Yes	Yes	Yes	Severe hemiparesis
JA	L: 16, R: 30	2	No	No	–	No	No impairment
TL	L: 14, R: 30	15	Yes	No	Yes	Yes	Severe hemiparesis
MK	L: 1, R: 29	54	–	–	Yes	–	Mild hemiparesis
LG	L: 11, R: 28	39	Yes	Yes	Yes	Yes	Severe hemiparesis

**Table 2:** Individual patient performance pre- and post prism adaptation (PA) in the chimeric/non-chimeric face discrimination task. Number of ‘chimeric’ or ‘non-chimeric’ items correctly classified is indicated separately, each out of 20 stimuli given per class.

	Chimeric		Non-chimeric	
Before PA	After PA	Before PA	After PA
EY	4	20	17	14
AM	12	18	16	18
PH	8	9	12	13
BH	8	10	16	18
MK	1	9	19	15
LG	2	6	12	16
